# Maggot protein ameliorates dextran sulphate sodium-induced ulcerative colitis in mice

**DOI:** 10.1042/BSR20181799

**Published:** 2018-11-28

**Authors:** Rong Wang, Lei Wang, Yongzheng Luo, Daojuan Wang, Ronghui Du, Jiancheng Du, Yong Wang

**Affiliations:** 1 State Key Laboratory of Analytacal Chemistry for Life Science and Jiangsu Key Laboratory of Molecular Medicine, Medical School, Nanjing University, Nanjing 210093, China; 2 School of Chemistry and Life Sciences, Nanjing University Jinling College, Nanjing, 210089, China; 3 Jiangsu Yicheng Bio Technology Co., Ltd., Nantong 226000, China

**Keywords:** dextran sulfate sodium, inflammation, maggot protein, mice, ulcerative colitis

## Abstract

Ulcerative colitis (UC) is a common chronic remitting disease but without satisfactory treatment. Maggots are known as a traditional Chinese medicine named as ‘wu gu chong’. The aim of the present study was to investigate the therapeutic effect of the maggot protein on dextran sulphate sodium (DSS)-induced colitis in C57BL/6 mice. In the present study, female C57BL/6 mice were given sterile water containing 3% DSS to establish the model of UC. Mice were randomly divided into five groups: control group (sterile water), model group (DSS), treatment group (DSS + maggot protein), mesalazine group (DSS + mesalazine), and maggot protein group (sterile water + maggot protein). The mental state, defecate traits, and changes in body weights were recorded daily. The disease activity index (DAI) as a disease severity criterion was calculated based on body weights and stool consistency and bleeding. All the mice were killed on the 12th day. Colon length, colon histological changes, and other inflammatory factors were analyzed and evaluated. The results showed that colitis models of mice were established successfully. Administration of maggot protein markedly suppressed the severity of UC compared with the DSS model group. Furthermore, maggot protein potently ameliorated DSS-induced weight loss, colon shortening, and colon histological injury. Moreover, the maggot protein exerted anti-inflammatory effects via inhibition of the activation of the nuclear factor κB (NFκB) signaling pathway. In summary, treatment by maggot protein was able to improve not only the symptoms of colitis, but also the microscopic inflammation in mice with DSS-induced colitis. The present study may have implications for developing an effective therapeutic strategy for inflammatory bowel diseases (IBDs).

## Introduction

Ulcerative colitis (UC) is one of the most common chronic inflammatory bowel diseases (IBDs), which is characterized by dysfunction of the innate and adaptive immunity, resulting in colonic mucosal injuries and histological changes in intestines manifested by body weight loss, altered stool consistency, bloody feces, and colonic shortening [1–3]. The disease pathology is caused by a combination of environmental factors, genetic specificity, and intestinal flora imbalance [4]. Although the exact mechanisms for the pathogenesis of UC are still unclear, it is widely thought to result from dysfunction of the mucosal immune responses [5]. Dextran sulphate sodium (DSS)-induced colitis is one of the well-established mouse models that have been shown to simulate human UC pathology [6–8], and recent preclinical studies have supported this colitis model as a system to evaluate the role of inflammation in UC using the inflammatory index as a readout [9].

Insects are a large, unexplored, and unexploited source that may supply potentially beneficial compounds for medication [10]. Maggots, known as a traditional Chinese medicine ‘wu gu chong’, are the larvae of *Lucilia sericata*. Housefly maggots have been used clinically to cure individuals suffering from ecthyma, wounds, and bacterial infection of the digestive organs since the Ming Dynasty (1368 A.D.) in China [11,12]. When combined with other drugs, the maggot protein demonstrates beneficial effects on coma and gastric cancer as well [11]. Maggot therapy is the fastest and most-efficient debridement therapy in wounds with necrotic tissue and infection, which has strong antimicrobial activity [13]. Recent studies have shown that maggot excretions/secretions are able to suppress multiple neutrophil pro-inflammatory responses, which include degranulation, chemotaxis, respiratory burst, and integrin expression. Interestingly, maggot excretions/secretions do not seem to affect the antimicrobial activities of neutrophils [14]. Mucins are glycoproteins that are commonly located on the surfaces of epithelial cells and they are assumed to mediate many interactions between these cells and their milieu [15,16]. Mucins provide a protective layer over the intestines. The expression of MUC1 and MUC2, two markers that are correlated to the amount of mucins, are decreased in UC [17,18]. Thus, mucin integrity is measured as one of the indicators of the severity of colitis [17]. Tight junction protein is another intestinal mucosal barrier, which is closely linked to intestinal permeability [19,20]. In UC, the tight junction protein is markedly destroyed. However, the maggot protein has been shown to be able to induce and promote remission [20].

IL-6, IL-8, and tumor necrosis factor α (TNF-α) are common pro-inflammatory cytokines which are significantly overexpressed in UC [21–23]. Consistently, the anti-inflammatory cytokine IL-10 is found to be down-regulated in UC models [24,25]. Recent research found that a cecropin-like peptide from maggot had antimicrobial activity against Gram-negative bacteria and immunomodulatory activities, which decreased the TNF-α production in human peripheral blood mononuclear cells (PBMCs) and induced cellular migration in human keratinocytes [26]. Our present study aimed to evaluate whether the addition of the maggot protein could protect mice with DSS-induced acute colitis. In the present study, we demonstrated that the maggot protein may ameliorate DSS-induced colitis by maintaining balanced immune responses through suppressing pro-inflammatory cytokines such as TNF-α and up-regulating IL-10 expression in colons and blood.

## Materials and methods

### Maggots and maggot powder preparation

Blowflies were maintained in cages under the standard conditions, and their larvae were collected. Next, maggots were transferred to a large pond filled with husks of wheat seeds, milk powder, and yeast extracts. After breeding, fresh maggots were washed three times with distilled water, heat-processed into dried maggots and then ground into powder. Finally, powder was sterilized with Cobalt 60 and vacuum packed.

### Reagents and experimental animals

DSS (MW: 36000−50000) was obtained from MP Biomedicals (OH, U.S.A.). All other reagents used were of analytical grade. Female, 6–8-week-old C57BL/6 mice weighing approximately 20 g were purchased from Qinglongshan (Nanjing, China). These mice were housed in specific pathogen-free (SPF) environment at a temperature of 22 ± 1°C, and with a relative humidity of 50 ± 1%, and a light/dark cycle of 12/12 h. Free access to food and water was provided. The experiments were conducted according to institutional animal ethics guidelines.

### Induction and treatment of experimental colitis

Experimental colitis was induced by continuous administration of DSS (3% w/v) in drinking water to C57BL/6 mice for 7 days [27,28]. For treatment with the maggot protein, mice received this protein with a daily dose of 1 g/kg (w/w) through an intragastric cannula for 12 consecutive days starting on the same day as DSS was delivered. Alternatively, mice were similarly given the positive control drug masalazine, an aminosalicylate anti-inflammatory drug commonly used to treat IBDs including UC, at 200 mg/kg (w/w) as a treatment regimen (*n*=5 for each group). The behavior of mice, body weight, and stool consistency were observed and recorded on a daily basis. After 12 days, all mice were killed and their colons and serum were collected for further study.

### Disease activity index score, colon length, and colon histopathological analysis

DSS-induced mouse colitis was scored as the disease activity index (DAI) using the described criteria [29]. In brief, severity in body weight loss, stool consistency alteration and bleeding were scored. Weight losses of 0, 1–5, 5–10, 10–20, and >20% were scored as 0, 1, 2, 3, and 4, respectively. As for stool consistency, 0 was scored for normal well-formed particles, 1 for loose stools, 2 for semi-formed stools, 3 for liquid stools, and 4 for diarrhea. Bleeding was scored 0 for no blood, 1 for trace, 2 for mild hemoccult, 3 for obvious hemoccult, and 4 for gross bleeding. Next, these subscores were added and the sum was divided by three to obtain the DAI scores which ranged from 0 to 4.

Mice were killed by cervical dissociation, and colons from the cecum to the anus were cut and their length was measured [17]. Approximately 10% of the colon was fixed in 10% buffered formalin, paraffin embedded, and stained with Hematoxylin and Eosin (H&E) for histopathological examination [30]. The rest of the colons were stored in PBS for Western blot and quantitative real-time reverse-transcription PCR (RT-qPCR).

### Western blot analysis

Colon samples were excised and homogenized in RIPA lysis buffer (Beyotime Biotechnology, Shanghai, China) containing 1 mM Pierce™ phosphatase inhibitor (Thermo Scientific, CA, U.S.A.) and 0.1% Halt™ protease inhibitor cocktail (Thermo Scientific, CA, U.S.A.). The mixture was centrifuged (13000 rpm, 20 min, 4°C) and the supernatants were collected. Protein concentration was measured using Bradford’s colorimetric method. Next, the extractive was mixed with a 5× SDS/PAGE sample buffer. Equal amounts of proteins (30 μg) were separated by a 10% SDS/PAGE gel and then transferred to PVDF membranes (Merck Millipore). After blocking in a 5% BSA buffer for 1.5 h, membranes were incubated with respective primary antibodies (occludin, zonula occluden-1 (ZO-1), TNF-α, nuclear factor-κB (NFκB), IκB) (Cell Signaling Technology, MA, U.S.A.) at 4°C overnight. After washing with TBST three times, membranes were incubated with a secondary antibody (Bioworld, U.S.A.) for the target bands for 1.5 h at room temperature. For visualization of the bands, all membranes were incubated with the immobilon western chemiluminescent HRP substrate (Millipore, U.S.A.) for desired durations. The relative density of the band for the protein of interest was compared with the band for the housekeeping gene *GAPDH* in each group.

### ELISA

Blood samples were collected from eyes. The serum was separated immediately and stored at −20°C for further analysis. The concentrations of IL-6 and IL-10 were measured by ELISA kits (Elabscience Biotechnology, China) according to the manufacturer’s instructions.

### Colon mRNA extraction and RT-qPCR assay

Total RNA was extracted by using TRIzol (Beyotime Biotechnology, Shanghai, China) and the cDNA was synthesized by using a reverse transcription kit (Takara, Beijing, China) according to manufacturer’s instructions. The housekeeping gene *GAPDH* was used as an internal control. Mouse mRNA primer sequences for the target genes are listed in Table 1. RT-qPCR reactions were performed with an ABI Viia 7 system by using the SYBR® Green PCR master mix (Takara, Beijing, China). The relative mRNA concentrations were calculated by E = 2^−ΔΔ*C*^_t_ and the critical threshold cycle (*C*_T_) value was measured in each reaction.

**Table 1:** Sequences of primers designed for RT-qPCR

Gene	Forward sequence	Reverse sequence
*TNF-α*	TCTCCAGCCACCAGCCCTCTAA	TGGCCATGGTAGGAGAAACAGG
*IL-8*	CACCCTCTGTCACCTGCTCAA	ATGGCGCTGAGAAGACTTGGT
*MUC1*	TCTCCAGCCACCAGCCCTCTAA	TGGCCATGGTAGGAGAAACAGG
*MUC2*	GGGAGGGTGGAAGTGGCATTGT	TGCTGGGGTTTTTGTGAATCTC

### Statistical analysis

All data are expressed as the mean ± S.D. from at least three independent experiments. The difference between two groups was evaluated by the Student’s *t* test. ANOVA was used to compare amongst three or more groups. *P*-values of 0.05 or less were considered statistically significant. All analysis was performed with GraphPad Prism software (San Diego, CA, U.S.A., version 6.07).

## Results

### Improvement of the DAI in mice treated with DSS by the maggot protein

**Figure 1:**
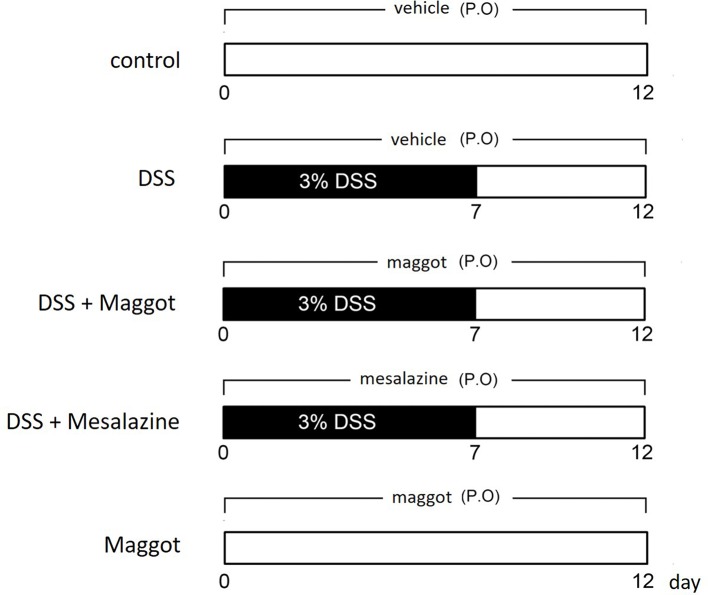
A schematic diagram indicating the experimental design All mice were randomly divided into five groups: Control mice that only received sterile water (Control); mice that received 3% DSS dissolved in drinking water for the first 7 days for creating the mouse colitis model (DSS); mice that orally received a daily administration of 1 g/kg (w/w) maggot protein through an intragastric cannula for the entire 12-day period (DSS + maggot protein); mice that orally received a daily administration of 200 mg/kg (w/w) of mesalazine through an intragastric cannula for the entire 12-day period (DSS + mesalazine); and mice that only received the maggot protein (sterile water + maggot protein).

**Figure 2:**
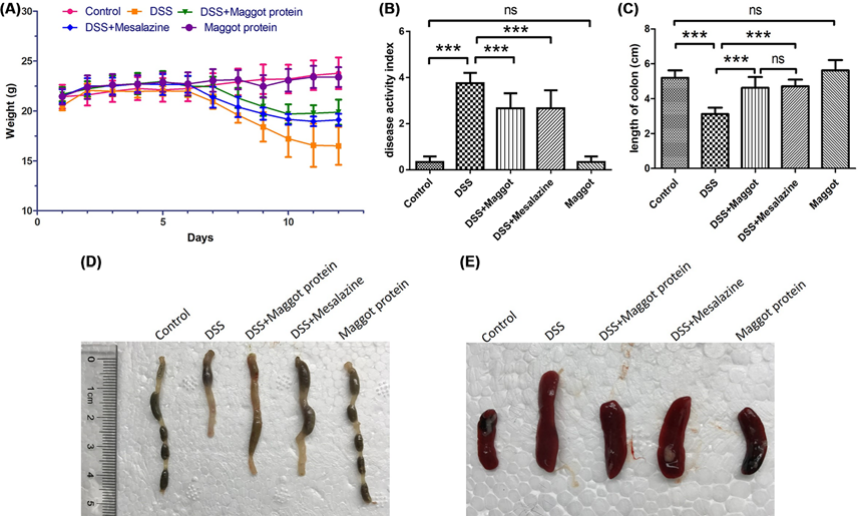
Effects of the maggot protein on the DAI score and the colon length of mice with DSS-induced colitis Mice were treated as explained in Figure 1. (**A**) Mouse body weights were recorded daily and shown. (**B**) The DAI represented the average score of clinical symptoms including the loss of body weight, abnormal stool consistency, and bleeding. (**C**,**D**) The colons of each mouse were harvested and their length was measured. The data were expressed as the mean ± S.D.; ***P<0.001, ns: no significance. (**E**) Mouse spleens were collected and displayed.

Mice were treated as indicated in Figure 1 for setting up the colitis model. The loss of body weight, stool consistency, and bleeding are all significant symptoms of DSS-induced colitis. We observed that loss of body weight in mice treated with DSS appeared from the sixth day. Similar to mesalazine, the commonly used anti-inflammatory drug for treating UC, the maggot protein could robustly ameliorate the loss of body weight (Figure 2A). Compared with the mice that only received water, the DAI score, an index of the severity of colonic inflammation was increased in DSS-exposed mice, and administration of the maggot protein evidently improved these symptoms (Figure 2B). The colon length was another reproducible and indirect indicator for the colitis severity of intestinal inflammation [31]. The administration of DSS induced a significant reduction in colon length when compared with control mice (Figure 2C,D). The supplementation of the maggot protein also showed a significant reversal on reducing colon shortening compared with mice treated with DSS. It was clear that the maggot protein and mesalazine had similar effects in DSS-induced mouse colitis. It was also observed that DSS led to spleen enlargements accompanied by inflammatory changes (Figure 2E). However, treatment with the maggot protein or mesalazine was able to suppress DSS-induced spleen enlargement.

### Histopathological analysis of DSS-induced acute colitis

**Figure 3:**
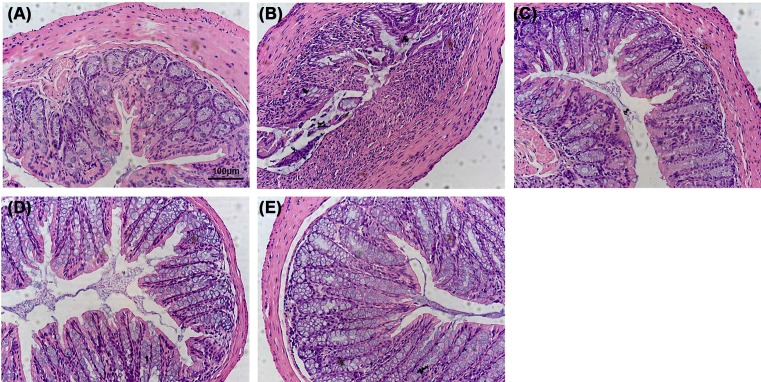
Effects of the maggot protein on the histopathological features in DSS-induced mouse colitis Mice were treated as explained in Figure 1. (**A**) control group (sterile water), (**B**) model group (DSS), (**C**) treatment group (DSS + maggot protein), (**D**) mesalazine group (DSS + mesalazine), (**E**) maggot protein group (sterile water + maggot protein). Colon was fixed in 10% buffered formalin for histopathological examination, paraffin embedded, and stained with H&E. Representative images of the colonic tissues were displayed. Scale bar = 100 μm.

The colon of UC revealed a large number of histological lesions, which included loss of crypts, edema, mucosal erosions, and the infiltration of inflammatory cells in the mucosa [32,33]. Compared with control mice (Figure 3A), mice treated with DSS showed serious injuries of colon mucosa, loss of histological structures, strong epithelial erosion, a marked decrease in the number of crypts, and pronounced infiltration of inflammatory cells (Figure 3B). In contrast, mice treated with the maggot protein (Figure 3C) exhibited reduced symptoms of acute UC inflammation with a similar efficacy as mesalazine (Figure 3D). Mice that only received the maggot protein demonstrated no overt histological changes (Figure 3E).

### Tight junction protein expression and NFκB signaling pathway activation in the colon of mice with DSS-induced colitis

**Figure 4:**
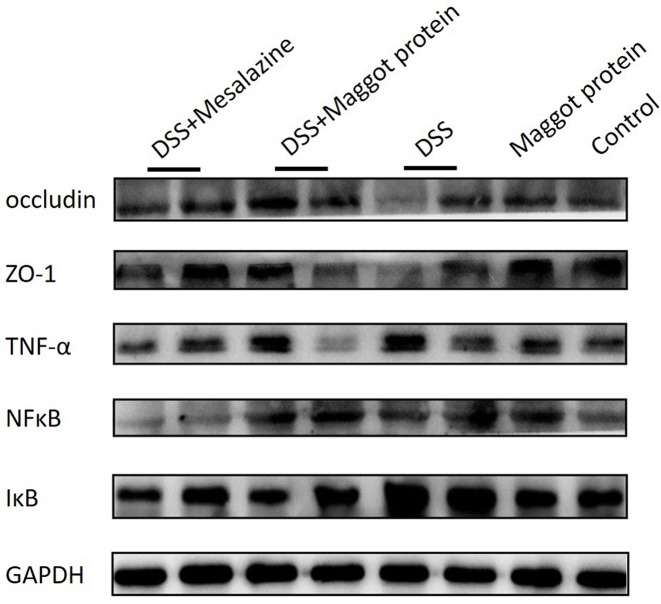
Effects of the maggot protein on the expression of the tight junction proteins and the activation of the NFκB signaling pathway in DSS-induced mouse colitis Mice were treated as explained in Figure 1. Total colon tissue protein was extracted for measuring the expression of pro-inflammatory cytokines and NFκB signaling proteins by Western blot with antibodies specific for occludin, ZO-1, TNF-α, NFκB, and IκB. The housekeeping gene *GAPDH* was used as a protein loading control.

To figure out the mechanisms that may account for the therapeutic effects described above, we measured the protein expression levels of the pro-inflammatory cytokines in the intestinal tissue of mice treated with the maggot protein after DSS administration by Western blot (Figure 4). Mice with DSS-induced colitis exhibited substantially increased expression of TNF-α, NFκB, and IκB, compared with naïve mice. However, expression of these proteins in mice with DSS-induced colitis was markedly down-regulated following treatment with the maggot protein or mesalazine. Increased expression of NFκB and IκB in mice the DSS-induced colitis suggested mobilization of the NFκB signaling pathway by DSS, and the maggot protein could inhibit such pro-inflammatory factors. Furthermore, expression of occludin and ZO-1 protein in mice treated with DSS was clearly decreased as a result of acute colitis. The maggot protein and mesalazine were capable of up-regulating these tight junction proteins after the induction of colitis by DSS.

### The concentration of pro-inflammation cytokines in the serum of DSS-induced colitis mice

**Figure 5:**
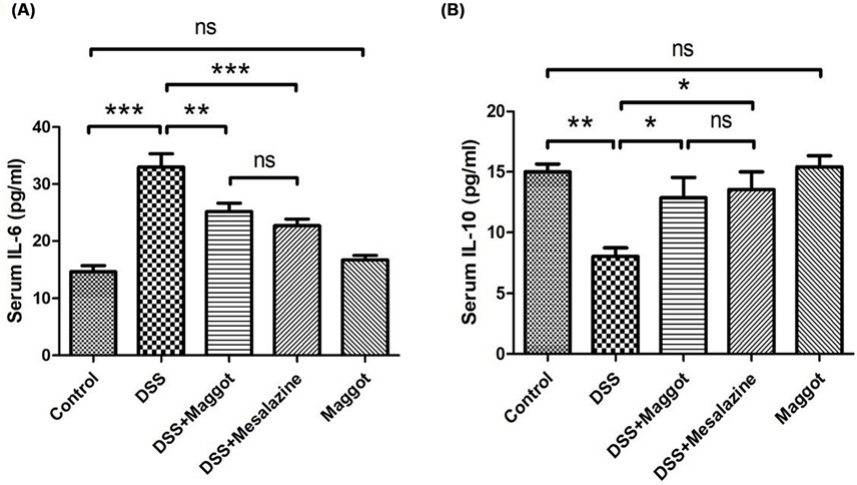
Effects of the maggot protein on the regulation of serum pro-inflammatory cytokines mice with DSS-induced colitis Mice were treated as explained in Figure 1. Blood samples were collected retro-orbital bleeding and examined by ELISA. (**A**) Serum levels of IL-6 and (**B**) IL-10 were measured. Data are expressed as the mean ± S.D. *P<0.05, **P<0.01, ***P<0.001.

As the maggot protein has been shown to play a potent negative regulatory role in pro-inflammatory responses, we next undertook to determine the serum levels of pro-inflammatory cytokines by ELISA. The results demonstrated that serum IL-6 in mice treated with DSS was substantially increased (Figure 5A). However, such enhanced levels of serum IL-6 were suppressed by maggot or mesalazine. The maggot protein had no regulatory effect on naïve mice (Figure 5A). As for serum IL-10, DSS-treated mice showed a marked decrease (Figure 5B). However, maggot protein and masalazine could restore the basal levels of IL-10 in serum (Figure 5B). Similarly, the maggot protein had no effect on the expression of serum IL-10 in naïve mice.

### The mRNA levels of the pro-inflammatory cytokines and mucins in the colon of mice with DSS-induced colitis

**Figure 6:**
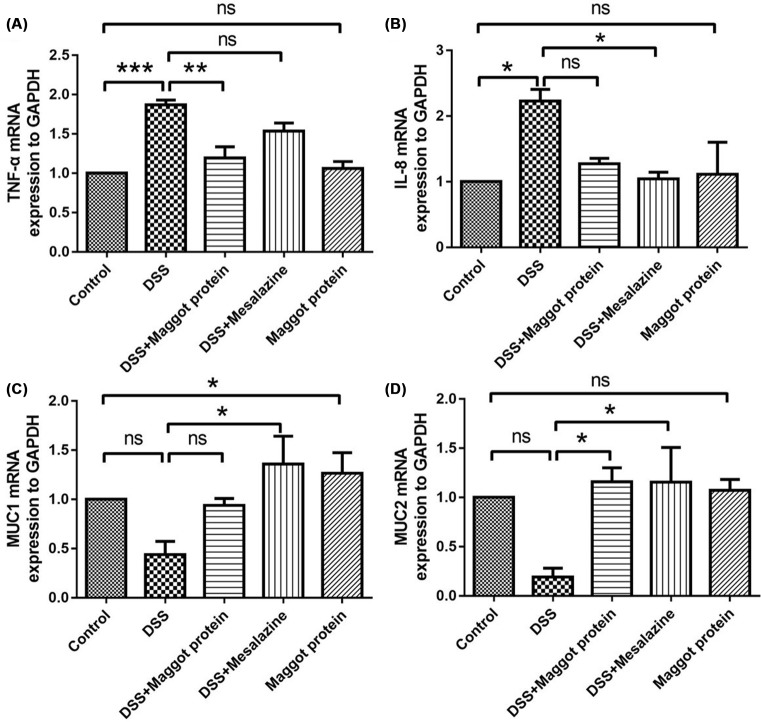
Effects of the maggot protein on the mRNA levels of the pro-inflammatory cytokines in the colon of mice with DSS-induced colitis Mice were treated as explained in Figure 1. Mouse colon mRNA was extracted for determining the mRNA abundance of TNF-α (**A**), IL-8 (**B**), MUC1 (**C**), MUC2 (**D**) by RT-qPCR. The housekeeping gene *GAPDH* was used as a loading control. The mRNA expression levels of the cytokines and mucins in the mice that received various treatments were normalized to the levels in naïve control mice which were set as 1. Data are expressed as the mean ± S.D.; *P<0.05, **P<0.01, ***P<0.001, ns: no significance.

We next measured the mRNA levels of these pro-inflammatory cytokines and mucins in the colon by RT-qPCR. Both TNF-α and IL-8 transcripts were substantially up-regulated in mice with DSS-induced colitis, and treatment with the maggot protein was able to reverse the enhancement, although the maggot protein-mediated suppression did not reach a statistical significance (Figure 6A,B). Treatment with mesalazine also suppressed the up-regulated cytokine transcript levels or at least demonstrated a trend of suppression. mRNA expression of these two cytokines in the colon of naïve mice treated with the maggot protein alone was not modulated compared with the naïve control mice. In addition, we observed that mice with DSS-induced colitis had decreased levels of MUC1 (Figure 6C) and MUC2 (Figure 6D) at the mRNA level. Interestingly, mice treated with either the maggot protein or mesalazine restored the mRNA levels of these two mucin proteins to the basal levels or even higher than the basal levels (Figure 6C,D). Again, treatment of naïve mice with the maggot protein alone did not alter the mucin levels.

## Discussion

UC is a recurrent and prolonged inflammatory disease common in digestive system. Its pathological manifestations are diverse, including diarrhea, abdominal pain, and tenesmus etc. In recent research, a cecropin-like peptide from maggot had antimicrobial activity against Gram-negative bacteria and immunomodulatory activities [26]. We speculated that one peptide responsible for antibacterial activity [34] had anti-inflammatory effects on IBD. Our present study demonstrated that the maggot protein could suppress DSS-induced colitis in mice and improve acute colonic injuries. Furthermore, treatment with the maggot protein could inhibit pro-inflammatory responses, indicating that the maggot protein may have a potent anti-inflammatory effect on UC, which was demonstrated by the restoration of the DAI scores. Notably, treatment with the maggot protein was able to reduce losses of crypts, edema, and mucosal erosions in the mucosa determined by the histopathological analysis. It has been reported that DSS can induce splenomegaly accompanied by inflammatory changes [32]. Increased colonic infiltration of T cells, neutrophils, and macrophages is involved in DSS-induced mouse colitis [33,35]. The maggot protein is thus predicted to be able to inhibit the infiltration of T cells, neutrophils, and macrophages to ameliorate DSS-induced acute colitis. Although we have not analyzed the splenocytes at the individual cellular level, we did observe that treatment with the maggot protein was able to suppress the DSS-induced splenomegaly. In our study, normal mice treated with the maggot protein did not show any abnormalities and protein did not have any toxic effects on the spleen.

Given the capacity of the maggot protein in suppressing UC, we next tried to investigate the putative mechanisms that may account for the therapeutic effect of this protein. Although the exact underlying mechanism of UC is still unknown, the NFκB signaling pathway has been widely believed to be able to mediate and maintain remission in UC [36,37], which is also supported by our data. NFκB, a redox-sensitive transcription factor, is a pivotal regulator of inflammation, innate immunity, and tissue integrity [38,39]. NFκB mediates pro-inflammatory responses by up-regulating the infiltration of neutrophils and macrophages, inducing a self-sustaining phlogogenic loop [38]. Most likely, regulation of NFκB is a key component of the anti-inflammatory activity of the maggot protein. It has previously been noted that tight junction proteins are markedly destroyed in UC [40,41]. Consistently, our data showed that the expression of occludin and ZO-1 was decreased notably in DSS-induced mouse colitis. The maggot protein could markedly restore expression of occludin and ZO-1 in mice with DSS-induced colitis. Furthermore, it has been reported that the DSS-induced colitis model is characterized by increased serum levels of IL-6, IL-17, and TNF-α and decreased levels of IL-4 and IL-10 [25,42]. Again, we also demonstrated that the maggot protein could markedly restore the serum expression levels of IL-6 and IL-10 in mice treated with DSS. In all our comparisons, the immunosuppressive activity of the maggot protein was similar to the positive control drug mesalazine.

Next, in order to further verify the anti-inflammatory effect of the maggot protein, we monitored some of the inflammatory cytokines and mucins at the mRNA level. These data are consistent with the conclusion that the maggot protein is capable of suppressing DSS-mediated UC, supporting its therapeutic effect. Mucins may contribute to the barrier effect between gut mucosa and their milieu [16,18,43].

In conclusion, our present study demonstrated that the maggot protein could ameliorate the symptoms of UC induced by DSS, resulting in an overall improvement in both macroscopic and histological parameters. The immunosuppressive potential of the maggot protein may depend on the NFκB signaling pathway which plays an important role in the regulation of immune cell infiltration and inflammatory cytokine expression. However, the specific mechanism of the amelioration of UC by the maggot protein is still unclear, which warrants further experiments for clarification. Our study provides a potential therapeutic strategy for treating UC by exploiting the anti-inflammatory effect of the maggot protein.

## Abbreviations

DAI: disease activity index
DSS: dextran sulphate sodium
GAPDH: glyceraldehyde 3-phosphate dehydrogenase
HRP: horseradish peroxidase
IκB: inhibitor of NF-κB
IBD: inflammatory bowel disease
IL: interleukin
MUC: mucin
MW: molecular weight
NFκB: nuclear factor κB
RIPA: radio-immunoprecipitation assay
RT-qPCR: quantitative real-time reverse-transcription PCR
TNF-α: tumor necrosis factor α
UC: ulcerative colitis
ZO-1: zonula occluden-1
